# GA3-Induced *SlXTH19* Expression Enhances Cell Wall Remodeling and Plant Height in Tomatoes

**DOI:** 10.3390/plants13243578

**Published:** 2024-12-21

**Authors:** Junfeng Luo, Xi Wang, Wenxing Pang, Jing Jiang

**Affiliations:** 1 College of Horticulture, Shenyang Agricultural University, Shenyang 110866, China; 2022220353@stu.syau.edu.cn (J.L.); 2021200112@stu.syau.edu.cn (X.W.); 2 Key Laboratory of Protected Horticulture of Education Ministry, Shenyang 110866, China

**Keywords:** tomato, *SlXTH19*, GA, plant height, cell wall

## Abstract

Plant height represents a pivotal agronomic trait for the genetic enhancement of crops. The plant cell wall, being a dynamic entity, is crucial in determining plant stature; however, the regulatory mechanisms underlying cell wall remodeling remain inadequately elucidated. This study demonstrates that the application of gibberellin 3 (GA3) enhances both plant height and cell wall remodeling in tomato (*Solanum lycopersicum* L.) plants. RNA sequencing (RNA-seq) results of GA3 treatment showed that the DEGs were mostly enriched for cell wall-related pathways; specifically, GA3 treatment elicited the expression of the cell wall-associated gene *XYLOGLUCAN ENDOTRANSGLUCOSYLASE/HYDROLASE 19* (*SlXTH19*), whose overexpression resulted in increased plant height. Comparative analyses revealed that *SlXTH19*-overexpressing lines exhibited larger cell dimensions and increased XTH activity, along with higher contents of lignin, cellulose, and hemicellulose, thereby underscoring the gene’s role in maintaining cell wall integrity. Conversely, treatments with ethephon (ETH) and 1-Naphthaleneacetic acid (NAA) led to suppressed plant height and reduced *SlXTH19* expression. Collectively, these findings illuminate a competitive interplay between GA and ethylene/auxin signaling pathways in regulating cell wall remodeling via SlXTH19 activation, ultimately influencing tomato plant height.

## 1. Introduction

Plant height is an important agronomic trait that is closely linked to yield potential [1]. It can affect crop architecture, water and fertilizer management, crowding tolerance, and mechanical harvesting, which in turn affect the economic benefits and yield of crops [2]. The genes encoding gibberellic acid (GA), auxin (IAA), brassinosteroid (BR), and strigolactone (SL) biosynthetic or signaling pathways have been shown to regulate plant height [3,4,5,6]. Among all phytohormones, GAs are regarded as the main factor in plant height determination [7]. Moreover, GAs also participate in the regulation of plant height through interactions with other phytohormones, such as IAA, BRs, SLs, cytokinin, and ethylene (ETH). In rice (*Oryza sativa*), low BR induces the GA biosynthesis-related gene *GA3ox2* and inhibits the GA catabolic gene *GA2ox3* to promote cell elongation [8]. Overexpression of *SlERF.H5* or *SlERF.H7* resulted in severe dwarf plants with lower gibberellin contents in tomatoes by repressing the expression of the gibberellin biosynthesis gene *SlGA20ox1* [9]. The BR, ET, and GA signals crosstalk also regulate plant development [10,11]. The cytokinin signaling pathway regulator SlRR6 regulates plant height through crosstalk with the GA and IAA pathways [12].

Plant height largely depends on cell length, which is regulated by the biosynthesis and expansion of the cell wall [13,14]. The cell wall comprises a complex network of carbohydrate polymers, lignin, and structural proteins [15]. The xyloglucan endotransglucosylases/hydrolases (XTHs) cleave and reconnect xyloglucan molecules in the primary cell wall to mediate cell wall remodeling [16,17]. PcBRU1, a member of the XTH family in pears (*Pyrus communis*), promotes stem growth and loosens the cell wall to improve plant height [18]. Overexpression of *PlXTH4* enhances stem strength and plant height by modulating secondary cell wall thickness in *Paeonia lactiflora* [19]. Exogenous BR treatment upregulates the expression of *AtXTH19* and *AtXTH23* via BES1 to regulate lateral root development under salt stress [20]. In maize (*Zea mays* L.), JA inhibited *ZmXTH1* expression by attenuating BR signaling, thereby repressing cell elongation and plant height [14]. In tomatoes, 37 SlXTHs were identified by analyzing whole genome sequences [21]. SlXTH5 is capable of modulating cell wall structure, and xyloglucan in particular, during fruit ripening [22]. However, the function of XTHs in determining plant height in tomato plants remains unknown.

In this study, we demonstrated that SlXTH19 is an important factor that regulates tomato plant height via the induction of cell elongation and cell wall remodeling and that GA enhances plant height by increasing *SlXTH19* expression levels through crosstalk with ETH and auxin signaling. Our study probes the role of xyloglucan endotransglucosylases/hydrolases in mediating GA-induced growth of tomatoes and lays the foundation for further study of the role of xyloglucan endotransglucosylases/hydrolases in tomatoes to assist in tomato breeding programs for novel architectural germplasm.

## 2. Results

### 2.1. GA Treatments Promote Cell Wall Remodeling and Plant Height

**Figure 1:**
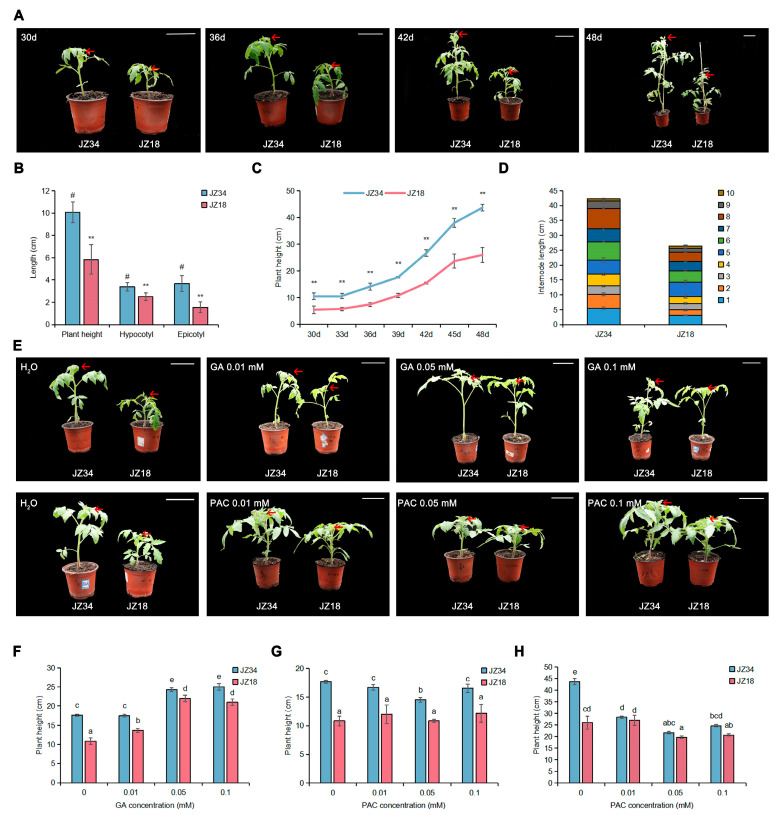
JZ34 seedlings have a taller phenotype than JZ18 seedlings. (**A**) Phenotypes of JZ34 and JZ18 at 30 d, 36 d, 42 d, and 48 d after seed breaking. Scale bars, 8 cm. (**B**) Plant height, hypocotyl length, and epicotyl length of 30-day-old JZ34 and JZ18 plants. (**C**) Plant heights of the JZ34 and JZ18 plants during different development stages. (**D**) Internode lengths of 30-day-old JZ34 and JZ18 plants. (**E**) Phenotypes of JZ34 and JZ18 plants sprayed with different concentrations of exogenous GA and paclobutrazol (PAC) for 9 d. Scale bars, 6 cm. (**F**) Plant heights of JZ34 and JZ18 plants after GA treatment for 9 d. (**G**) Plant heights of JZ34 and JZ18 plants after PAC treatment for 9 d. (**H**) Plant heights of JZ34 and JZ18 plants after PAC treatment for 18 d. Values represent the means ± SD, n = 3 pools, with 5 plants per pool. Student’s *t*-test was performed for (**B**,**C**); asterisks indicate significant differences compared with JZ34 (#) at ** *p* < 0.01. Different letters indicate significant differences (*p* < 0.05) according to Duncan’s test for (**F**–**H**).

To investigate the basis for the differences in plant growth between JZ34 and JZ18, we first compared plant height, hypocotyls, and epicotyls in JZ34 and JZ18 at 30 d. We found that plant height, hypocotyls, and epicotyls were greater in JZ34 than in JZ18 (Figure 1A,B). Compared to JZ34, the plant height of JZ18 was lower at different developmental stages (Figure 1C). The internode length of JZ18 was significantly shorter than that of JZ34 at 48 d, except for the third, fifth, and tenth internodes (Figure 1D). The fact that GA signals have a positive effect on plant growth led us to ask whether GA treatment could rescue the height defects in JZ18 plants. Different concentrations of GA (0.01, 0.05, and 0.1 mM) were applied to JZ34 and JZ18 tomato plants for 9 d to investigate the effect on plant growth (Figure 1E). Our results showed that GA effectively increased plant height in JZ18 and JZ34 (Figure 1F). The height difference between JZ34 and JZ18 was reduced by GA (Figure 1F). However, when the plants were treated with the GA biosynthesis inhibitor paclobutrazol (PAC), the height of the JZ34 seedlings decreased, whereas the JZ18 seedlings were unresponsive to this inhibitor (Figure 1E,G). We further treated JZ34 and JZ18 tomato plants with different concentrations of PAC for 18 d (Figure 1H). PAC treatment significantly inhibited the plant height of JZ18 after 0.05 and 1 mM PAC treatment; therefore, exogenous GA had a positive effect on tomato height.

**Figure 2:**
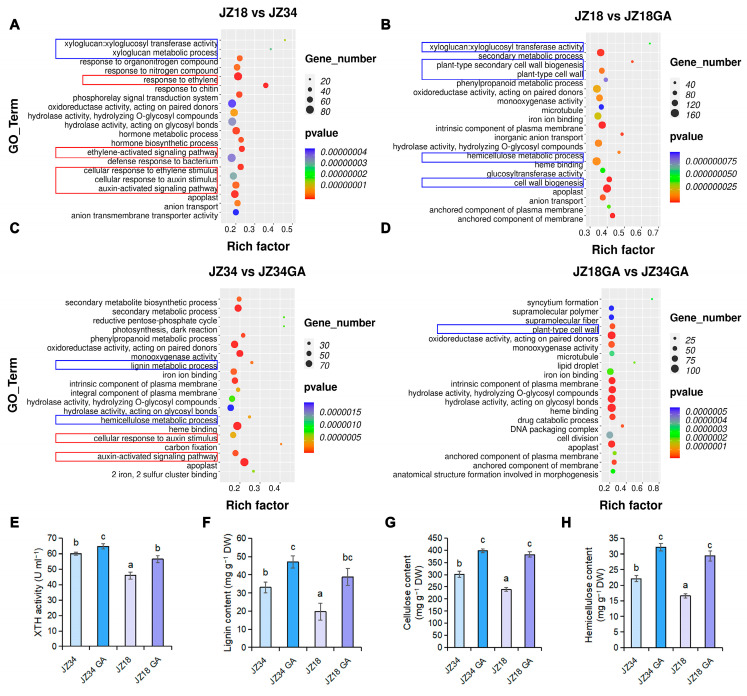
GA regulates XTH gene expression in tomato stems. (**A**–**D**) Gene ontology (GO) enrichment pathway scatterplot for the differentially expressed genes (DEGs) in JZ18 vs. JZ34, JZ18 vs. JZ18 GA, JZ34 vs. JZ34 GA, and JZ18 GA vs. JZ34 GA comparisons. DEGs screened with |log2(FC)| ≥ 1.0, *p* < 0.05, and a false discovery rate (FDR) < 0.05 among four pairwise comparisons. The blue boxes represent cell wall-related pathways and the red boxes represent ethylene and auxin-related pathways. (**E**–**H**) XTH activity and (**E**) lignin (**F**), cellulose, and (**G**) hemicellulose contents (**H**) in JZ34 and JZ18 without and with GA treatment. Values represent means ± SD, n = 3 pools, with 5 plants per pool. Different letters indicate significant differences (*p* < 0.05) according to Duncan’s test.

To identify the molecular pathways underlying plant height, we compared gene expression in the stems of JZ34 and JZ18 plants under GA treatment using RNA sequencing (RNA-seq). Gene ontology (GO) analysis showed that the DEGs were mostly enriched for cell wall-related pathways among the four comparisons (JZ18 vs. JZ34, JZ18 vs. JZ18-GA, JZ34 vs. JZ34-GA, and JZ18-GA vs. JZ34-GA), including xyloglucan metabolic process, xyloglucan/xyloglucosyl transferase activity, cell wall biogenesis, hemicellulose metabolic process, plant-type secondary cell wall biogenesis, plant-type cell wall, and lignin metabolic process (Figure 2A–D and Supplementary Table S1).

To explore the potential relationship between GA and the cell wall-related pathways, we examined the XYLOGLUCAN ENDOTRANSGLUCOSYLASE/HYDROLASE (XTH) activity and lignin, cellulose, and hemicellulose contents of JZ34 and JZ18 without and with GA treatment. XTH activity and lignin, cellulose, and hemicellulose contents of JZ34 were higher than those of JZ18 (Figure 2E–H). After GA treatment, XTH activity and lignin, cellulose, and hemicellulose contents were induced in both JZ34 and JZ18 (Figure 2E–H). These results provide evidence that GA promotes XTH activity and the accumulation of lignin, cellulose, and hemicellulose to regulate plant height.

### 2.2. SlXTH19 Displayed Stable and High Expression Levels During Tomato Stem Development in GA Treatment

**Figure 3:**
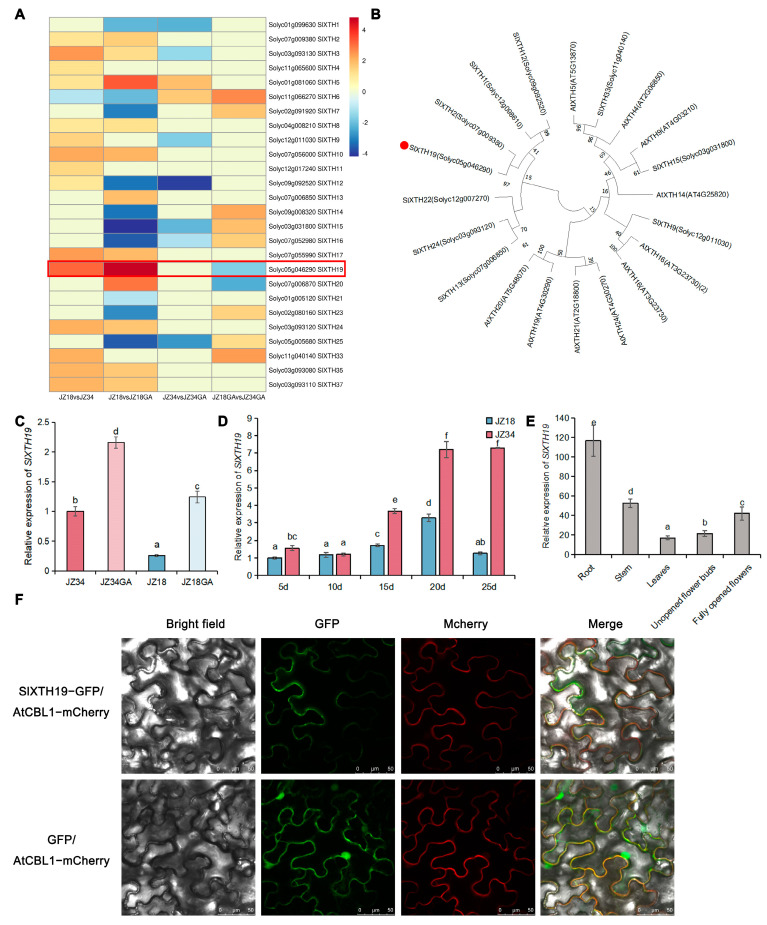
GA regulates *XTH* gene expression in tomato stems. (**A**) Heatmap representation of the expression of 26 candidate *SlXTH* genes. The red box represents the most significant difference. (**B**) Phylogenetic tree of XTH family members from *Solanum lycopersicum* and *Arabidopsis thaliana*. The red dot represents the SlXTH19. (**C**) Relative expression of *SlXTH19* in JZ34 (**A**) and JZ18 (**B**) plants under GA treatment for 9 d. (**D**) Relative expression of *SlXTH19* in JZ34 and JZ18 5 d, 10 d, 15 d, 20 d, and 25 d after seed breaking. (**E**) Tissue-specific expression of *SlXTH19*. (**F**) SlXTH19-GFP is localized to the plasma membrane of *Nicotiana benthamiana* cells. An mCherry-labeled plasma membrane marker (AtCBL1) was coexpressed to visualize the plasma membrane. Values represent means ± SD, n = 3 pools, with 5 plants per pool. Different letters indicate significant differences (*p* < 0.05) according to Duncan’s test.

Because XYLOGLUCAN ENDOTRANSGLUCOSYLASE/HYDROLASE (XTH) activity is higher after GA treatment, we first focused on the differential expression of *SlXTH* genes. The 26 XTH genes were differentially expressed between the stems of JZ34 and those of JZ18 under GA treatment (Figure 3A). *SlXTH19* was more highly expressed in JZ34 than in JZ18 and was hypersensitive to GA treatment in stems of JZ18 (Figure 3A). These results suggest that the increase in *SlXTH19* expression levels in the stems may be consistent with the change in plant height.

To confirm the effect of GA on the expression of *SlXTH19*, we performed RT-qPCR to measure the changes in *SlXTH19* expression in JZ34 and JZ18 stems after GA treatment. *SlXTH19* expression was significantly induced by GA in JZ18 and JZ34 for 9 d (Figure 3C). Taken together, these results suggest that SlXTH19 responds to GA to regulate plant height.

Phylogenetic analysis of the sequences further revealed that SlXTH19 was closely related to SlXTH2 (Figure 3B). However, SlXTH2 was not induced by GA, indicating that SlXTH19 and SlXTH2 may have different roles in plant growth and development. Studies on the expression pattern of *SlXTH19* at different stem growth stages were conducted. The expression of *SlXTH19* was induced in the stem of 15-day-old seedlings, and the expression of *SlXTH19* was higher in the stems of JZ34 than in those of JZ18 (Figure 3D). Studies on the expression pattern of *SlXTH19* in different tissues demonstrated its broad expression in plants, with the highest levels of transcripts in the roots and stems (Figure 3E). To investigate the cellular localization of SlXTH19 proteins, p35S:SlXTH19-eGFP fusion genes were transiently expressed in *N. benthamiana* leaf cells. As shown in Figure 3F, GFP fluorescence was detected in the cell membrane, indicating that the SlXTH19 protein was localized to the cell membrane.

### 2.3. Phenotypic Analysis of SlXTH19

**Figure 4:**
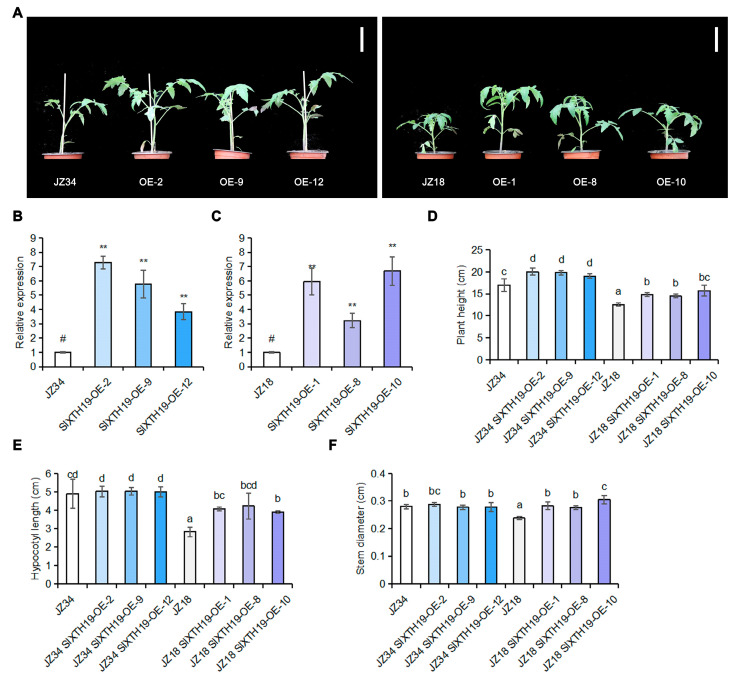
Overexpression of *SlXTH19* promotes tomato plant height. (**A**) Phenotypes of wild-type (JZ34 and JZ18) and *SlXTH19*-overexpressing transgenic plants are shown. Scale bars, 8 cm. (**B**,**C**) Relative expression of *SlXTH19* in *SlXTH19*-OE-JZ34 lines (**B**) and *SlXTH19*-OE-JZ18 lines (**C**). Values represent the means ± SD, n = 3 pools, with 5 plants per pool. Student’s *t*-test was performed; asterisks indicate significant differences compared with JZ34 and JZ18 (#) at ** *p* < 0.05, respectively. (**D**–**F**) Plant heights, hypocotyl lengths, and stem diameters of the plants shown in (**A**). Values represent means ± SD, n = 3 pools, with 5 plants per pool. Different letters indicate significant differences (*p* < 0.05) according to Duncan’s test.

To determine whether SlXTH19 is associated with plant height, we generated transgenic JZ34 and JZ18 plants overexpressing *SlXTH19* (Figure 4A). RT–qPCR analysis revealed three lines with high *SlXTH19* transcript levels: JZ18-*SlXTH19*-OE-1, JZ18-*SlXTH19*-OE-8, and JZ18-*SlXTH19*-OE-10, with *SlXTH19* levels approximately 3–6-fold higher than those of JZ18; and JZ34-*SlXTH19*-OE-2, JZ34-*SlXTH19*-OE-9, and JZ34-*SlXTH19*-OE-12, with *SlXTH19* levels approximately 3–7-fold greater than those of JZ34 (Figure 4B,C). The plant height, stem diameter, and hypocotyl length of three independent *SlXTH19*-overexpressing lines from JZ18 were significantly greater than those of the wild-type JZ18 (Figure 4D–F). In contrast, compared with JZ34, the plant height of the JZ34-*SlXTH19*-overexpressing line significantly increased. There was no difference in stem diameter or hypocotyl length between JZ34 and the JZ34-*SlXTH19*-overexpressing line (Figure 4D–F). These results indicate that SlXTH19 positively regulates plant height in tomatoes.

**Figure 5:**
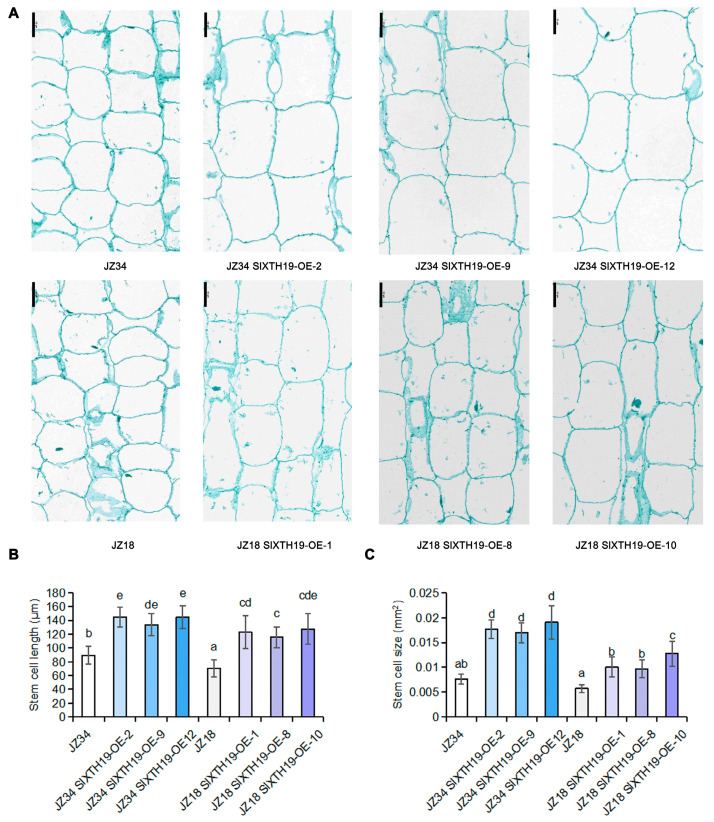
Overexpression of *SlXTH19* promotes tomato plant height by increasing cell length. (**A**) Images depicting photographs of the stem cells of different genotypes of *SlXTH19-OE* and wild-type (JZ34 and JZ18). Scale bars, 50 μm. (**B**,**C**) Stem cell length and cell size in the stems of *SlXTH19-OE* and wild-type (JZ34 and JZ18). The values represent the means ± SD, n = 3 pools, with 10 plants per pool. Different letters indicate significant differences (*p* < 0.05) according to Duncan’s test.

We also measured the cell length and cell size of the *SlXTH19*-OE and WT (JZ34 and JZ18) stems. Compared with those in JZ34 and JZ18, the stem cell length and size in the *SlXTH19*-OE stem were significantly greater (Figure 5A–C). Taken together, these results suggest that SlXTH19 promotes plant height development by regulating stem cell length.

### 2.4. Comparison of XTH Activity and Lignin, Cellulose, and Hemicellulose Contents

**Figure 6:**
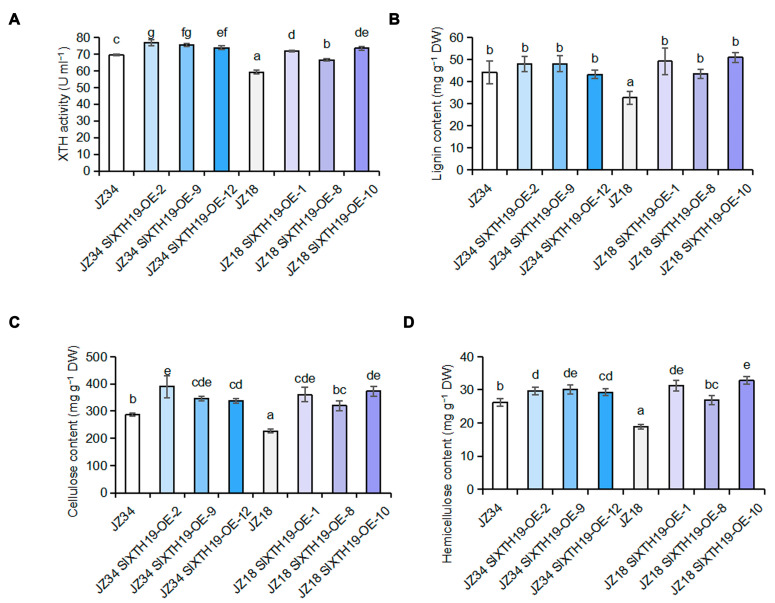
XTH activity and lignin, cellulose, and hemicellulose contents in *SlXTH19*-OE and wild-type (JZ34 and JZ18). The graph displays XTH activity (**A**), lignin content (**B**), cellulose content (**C**), and hemicellulose content (**D**) in both SlXTH19-OE and wild-type (JZ34 and JZ18). Values represent means ± SD, n = 3 pools, with 5 plants per pool. Different letters indicate significant differences (*p* < 0.05) according to Duncan’s test.

XTH activity and lignin, cellulose, and hemicellulose contents were examined in *SlXTH19*-OE (JZ34-*SlXTH19*-overexpressing and JZ18-*SlXTH19*-overexpressing) and WT (JZ34 and JZ18). XTH activity and cellulose and hemicellulose contents of JZ34-*SlXTH19*-overexpressing and JZ18-*SlXTH19*-overexpressing plants were significantly greater than those of JZ34 and JZ18 plants (Figure 6A,C,D). Compared with that in the WT plants (JZ18 and JZ34), the lignin content in the JZ18-*SlXTH19*-overexpressing plants significantly increased, while no difference was detected in the JZ34-*SlXTH19*-overexpressing plants (Figure 6B). These results indicate that SlXTH19 mediated cell wall remodeling to enhance plant height in tomatoes.

### 2.5. Response of Plant Height to Exogenous ETH and IAA Treatment in Tomatoes

**Figure 7:**
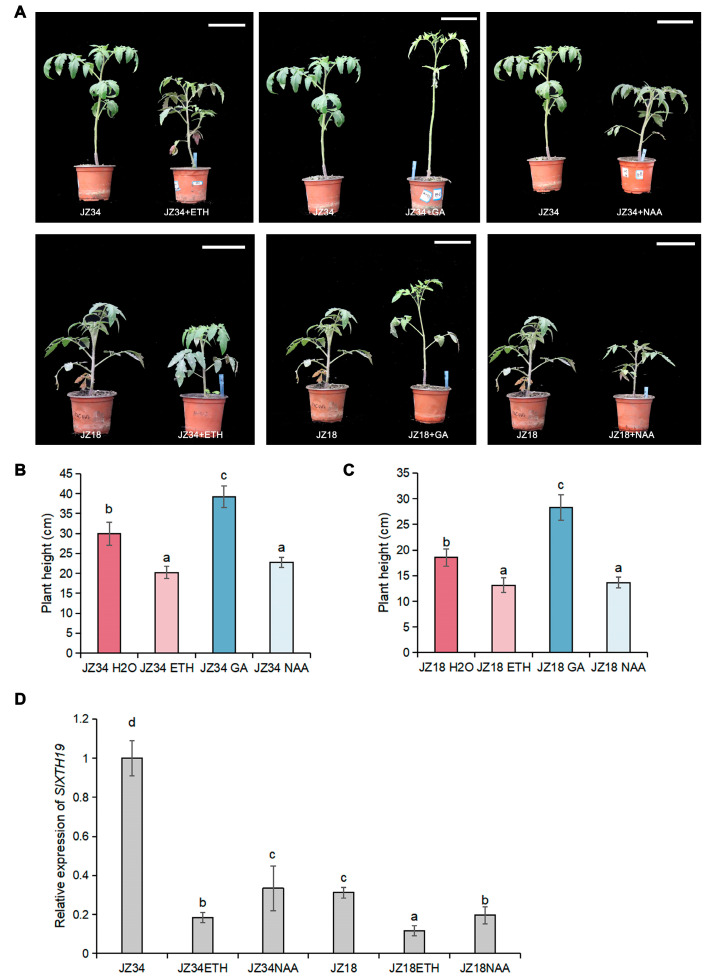
Effects of ETH, GA, and NAA treatments on plant height. (**A**) Phenotypes of JZ18 and JZ34 under ETH, GA, and NAA treatment for 20 d. (**B**,**C**) The plant heights of plants shown in (**A**). Scale bars, 8 cm. (**D**) Relative expression of *SlXTH19* under ETH and NAA treatments for 20 d. GA: 0.05 mM; ETH: 1 mM; NAA: 100 μM. The values represent the means ± SD, n = 3 pools, with 10 plants per pool. Different letters indicate significant differences (*p* < 0.05) according to Duncan’s test.

Previous studies have shown that the plant hormone pathway regulates plant height (Tong et al., 2014; Liu et al., 2023 [8,12]). GO pathway analysis revealed enrichment of DEGs in several ethylene and auxin pathways, including ethylene response, cellular response to auxin stimulus, auxin-activated signaling pathway, cellular response to ethylene stimulus, and ethylene-activated signaling pathway, indicating that ethylene and auxin pathways are involved in regulating the differences in plant height between JZ34 and JZ18 (Figure 2A). Therefore, we analyzed the effect of ethylene and auxin treatment on the heights of JZ34 and JZ18 plants. Treatment with ethylene and NAA significantly inhibited JZ34 and JZ18 plant heights compared to untreated JZ34 and JZ18 plants, contrary to GA treatment (Figure 7B,C).

The expression of *SlXTH19* was investigated in treated plants (Figure 7D). Compared with the controls, the expression levels of *SlXTH19* were upregulated by GA treatment (Figure 3C) and inhibited by ETH and NAA treatments (Figure 7D) in both JZ34 and JZ18 plants. These results further suggest that GA and ETH/NAA antagonistically regulate plant height via cell wall remodeling in tomatoes.

## 3. Discussion

Height is one of the most important agronomic traits for crops and affects plant architecture, dense planting, water and fertilizer management, and mechanical harvesting, which in turn affect the economic benefits and yield of crops [2]. In this study, we showed that spraying GA could increase the height of tomato plants (Figure 1), which is consistent with previous findings [6,23]. GA treatment increased XTH activity and lignin, cellulose, and hemicellulose contents (Figure 2E–H). Similarly, overexpressing *GA20ox1* increased the cellulose, lignin, and cell wall residues in maize plants [24]. In addition, GO analysis of transcriptome data from JZ34 and JZ18 plants under GA treatment showed that the DEGs were mostly enriched for cell wall-related pathways (Figure 2A–D). However, there are relatively few reports on GA-mediated cell wall remodeling to regulate plant height in tomatoes.

Cell wall relaxation and stretching are required for cell elongation and cell division, thereby regulating plant growth [25,26,27]. A candidate gene for cell wall remodeling, *SlXTH19*, was selected from the GA-treated JZ18 varieties and was significantly upregulated in GA-treated JZ18 seedlings (Figure 3A). In the promoter of *SlXTH19*, we found a GA response element P-box, which may result in the upregulated expression of *SlXTH19* after GA treatment of JZ18 plants. Similarly, GA enhanced the expression of *OsXTH8* and regulated the basal internodes of rice [28]. Here, *SlXTH19*-overexpressing plants exhibited increased plant heights and hypocotyl lengths due to an increase in cell length, suggesting that the expression of *SlXTH19* is required for plant height (Figure 4D,E, and Figure 5B). In addition, the larger cell size in *SlXTH19*-overexpressing plants resulted in increased stem diameter (Figure 4F and Figure 5C). XTH activity and lignin, cellulose, and hemicellulose contents were higher in *SlXTH19*-overexpressing plants than in JZ18 (Figure 6A–D), indicating that SlXTH19 mediated cell wall remodeling to enhance plant height. Overexpression of the cell wall-related gene *ZmXTH1* in maize plants promoted internode elongation [14]. Taken together, these findings support the notion that GA positively regulates SlXTH19-mediated cell wall remodeling to increase height in tomato plants.

In *Arabidopsis thaliana*, ethylene plays a negative role in the modulation of hypocotyl growth [29]. Two *Arabidopsis eto1* and *eto3* mutations increase the ethylene content and reduce hypocotyl length [30]. In a previous study, Populus ERF139 was shown to impact tree growth by coordinating xylem cell expansion and secondary cell wall deposition [31]. Overexpression of ZmPIN1a, which regulates auxin spatiotemporal asymmetric distribution, reduced plant height, internode length, and ear height [32]. In tomatoes, GA3 and IAA treatments resulted in the elongation of the far-red light-responsive internode, and XTH and EXP genes were linked to FR-induced internode elongation in tomatoes [33]. Similarly, shade light promoted stem growth and the expression of growth-related genes, including those involved in the metabolism of cell wall carbohydrates, and in auxin responses [34]. In this study, GO pathway analysis revealed enrichment of DEGs in the cell wall-related ethylene and auxin pathways (Figure 2A–D), suggesting that auxin and ethylene signaling are involved in cell wall remodeling to regulate plant height. Compared with the untreated plants, NAA and ETH treatments decreased the heights of JZ34 and JZ18 plants (Figure 7A–C). A recent study suggested that the JA mimic coronatine (COR)-mediated basic helix-loop-helix (bHLH) transcription factor ZmbHLH154 directly binds to the *ZmXTH1* promoter and inhibits basal internode elongation at the jointing stage (Wang et al., 2024 [14]). Here, exogenous NAA and ETH treatments repressed *SlXTH19* expression in JZ34 and JZ18 (Figure 7D), suggesting that ETH and NAA negatively regulate tomato plant height by inhibiting the expression of *SlXTH19*. In *A. thaliana*, GA promoted hypocotyl elongation by depending on ABA INSENSITIVE 4 (ABI4), which binds to *EXP2*, *XTH5*, and *XTH21* promoters and induces their expression [35]. Our results showed that GA and ETH/NAA antagonistically mediate plant height by regulating *SlXTH19* expression in tomatoes. 

## 4. Materials and Methods

### 4.1. Plant Materials and Growth Conditions

The fresh-market tomato high-generation inbred lines, JZ34 (long stem internode) and JZ18 (short stem internode) were developed by our group [36]. The JZ34, JZ18, and transgenic tomato plants were individually grown under the same growth conditions (25 °C, 16 h light, and 8 h dark). Shoot meristems were collected from JZ34 and JZ18 seedlings at 5 d, 10 d, 15 d, 20 d, and 25 d. The shoot meristems of transgenic tomato plants, JZ34, and JZ18 were collected at 30 d. All tissue samples were immediately frozen in liquid nitrogen and stored at −80 °C until use.

### 4.2. Exogenous Hormone Treatment

For the GA_3_ and paclobutrazol (PAC) treatments, 0.01, 0.05, and 0.1 mM GA_3_ (CG5571; Coolaber, Beijing, China) and PAC (CP8121; Coolaber) were sprayed on 30-day-old plants. For ETH and NAA treatment, 30-day-old JZ34 and JZ18 plants were treated with 1 mM ethephon (CE5121; Coolaber) [37] and 100 μM NAA (CN7541; Coolaber) [38], respectively. Treatment plants were sprayed (5 mL plant^−1^) and the control group was treated with an equal amount of deionized water. Each treatment included at least 30 seedlings with similar growth states. In this study, fresh stem samples were collected, frozen in liquid nitrogen, and stored at −80 °C until use.

### 4.3. Plasmid Construction and Plant Transformation

For overexpression experiments, the full-length cDNAs of SlXTH19 were amplified and introduced into the plant expression vector pCAMBIA3301 using the ClonExpress II One Step Cloning Kit according to the manufacturer’s manual (Vazyme, Nanjing, China). Agrobacterium-mediated transformation was performed as previously described (Wang et al., 2023 [36]).

### 4.4. Phenotype Observation

The phenotypes measured in this study included plant height, hypocotyl length, epicotyl length, internode length, and stem diameter. The plant height, hypocotyl length, epicotyl length, and internode length were measured directly with a ruler. The stem diameter was measured with a Vernier caliper. The upper, middle, and lower positions of each internode were measured, and the average value was taken as the stem diameter.

### 4.5. Cellulose, Hemicellulose, and Lignin Content Measurements

All the samples were dried in an oven at 80 °C to a constant weight and then ground to a fine powder. The supernatant liquid was used to determine the cellulase and hemicellulose contents, as described previously [39,40]. Lignin content was measured with a Lignin Content Assay Kit (Solarbio, Beijing, China).

### 4.6. Xyloglucan Endotransglucosyltransferase/Hydrolase (XTH) Activity Determination

XTH activity was measured by an ELISA test kit provided by Jiangsu Meimian Industrial Co., Ltd., Yancheng, China. Briefly, the sample (0.05 g) was added to a centrifuge tube (2 mL), and phosphate buffer (pH 7.2, 1 mL) was added and homogenized evenly with a homogenizer. After centrifugation for 20 min (3000 r/min), the supernatant was collected and used to determine activity. The XTH activity of the samples was determined according to the requirements of the XTH activity ELISA test kit.

### 4.7. Transcriptome Analysis

Three nodes from the GA-treated and untreated four-week-old JZ34 and JZ18 plants were collected for RNA isolation and RNA-seq analysis. An RNAprep Pure Plant Kit (DP441, Tiangen, Beijing, China) was used for the extraction of total RNA. The concentration and quality of the RNA were evaluated using a 2100 Bioanalyzer (Agilent Technologies, Palo Alto, CA, USA), and cDNA libraries were obtained and sequenced using the Illumina^NovaSeqTM^ 6000 system (LC Sciences, Houston, TX, USA). Then, the clean reads were mapped to the tomato reference genome (https://solgenomics.net/organism/Solanum_lycopersicum/genome (accessed on 8 April 2023)). Gene expression levels were determined using the RPKM (reads per kb per million reads) method. A false-discovery rate (FDR) of <0.05 was used to determine the *p*-value thresholds via multiple testing. All genes selected had a fold-change ≥ 1 and a *p* < 0.05. The differentially expressed genes (DEGs) were blasted against the GO database (http://amigo.geneontology.org/ (accessed on 8 April 2023)) to identify the pathways significantly associated with the DEGs. All the raw data have been deposited in the NCBI Sequence Read Archive under accession no. SUB14689245.

### 4.8. Subcellular Localization

The complete coding sequences of SlXTH19 and AtCBL1 without a stop codon were cloned and inserted into the pCAMBIA1302-GFP and pCAMBIA1300-mCherry vectors to generate the fusion expression vectors 35S::SlXTH19::GFP and 35S::AtCBL1::mCherry. Subsequently, the fusion constructs were transformed into *Agrobacterium tumefaciens* strain GV3101, which was subsequently infiltrated into *N. benthamiana* leaves. GFP fluorescence was observed using a confocal laser scanning microscope (Leica SP8, Wetzlar, Hessen, Germany) after 3 d. AtCBL1 was used as a plasma membrane marker. The fluorescence signal was observed as described previously [41].

### 4.9. Statistical Analysis

The data, including the means, standard deviations (SDs), and significant differences between samples, were analyzed using the SPSS 26.0 statistical program. The data are presented as the mean ± SD, and significant differences were compared using one-way ANOVA followed by Duncan’s test (*p* < 0.05) or independent Student’s *t*-tests, with *p* < 0.05 considered significant.

## 5. Conclusions

In this study, gibberellin 3 (GA3) treatment enhanced both plant height and cell wall remodeling in tomato plants. GA3 treatment elicited the expression of the cell wall-associated gene XYLOGLUCAN ENDOTRANSGLUCOSYLASE/HYDROLASE 19 (SlXTH19), whose overexpression resulted in increased plant height, XTH activity, along with higher contents of lignin, cellulose, and hemicellulose. Moreover, ethephon (ETH) and 1-Naphthaleneacetic acid (NAA) treatments led to suppressed plant heights and reduced SlXTH19 expression. Collectively, these findings illuminate a competitive interplay between GA and ethylene/auxin signaling pathways in regulating cell wall remodeling via SlXTH19 activation, ultimately influencing tomato plant height.

## Data Availability

The data are available from the corresponding authors upon reasonable request.

## Supplementary Materials

The following supporting information can be downloaded at: https://www.mdpi.com/article/10.3390/plants13243578/s1. Supplementary Table S1: The differentially expressed genes (DEGs) of Gene ontology (GO) enrichment pathway.
